# The CUSUM statistic of change point under NA sequences

**DOI:** 10.1007/s11766-021-4015-z

**Published:** 2021-12-22

**Authors:** Jin Ling, Xiao-qin Li, Wen-zhi Yang, Jian-ling Jiao

**Affiliations:** 1 School of Management, Hefei University of Technology, Hefei, 230009 China; 2 Center for Applied Mathematics, School of Mathematical Sciences, Anhui University, Hefei, 230061 China

**Keywords:** 62F12, CUSUM statistic, limit distribution, NA sequences, Brownian bridge

## Abstract

In this paper, we investigate the CUSUM statistic of change point under the negatively associated (NA) sequences. By establishing the consistency estimators for mean and covariance functions respectively, the limit distribution of the CUSUM statistic is proved to be a standard Brownian bridge, which extends the results obtained under the case of an independent normal sample and the moving average processes. Finally, the finite sample properties of the CUSUM statistic are given to show the efficiency of the method by simulation studies and an application on a real data analysis.

## §1 Introduction

Detecting a change-point and estimating its location are very important problems because of its extensive applications in many fields such as quality control, economics and finance, and so on. Many researchers pay attention to the study of change point detection. For example, Hsu [11] detected the shifts of parameter in gamma sequences; Bai [1] and Shi et al. [23] studied the mean shift models of change point; Kokoszka and Leipus [14] considered the CUSUM-type estimator for mean shift with dependent sequence; Lee et al. [15] and Na et al. [17] investigated the CUSUM statistic for parameter change in time series models; Horváth and Rice [10] summarized some classical methods in change point analysis; Christian et al. [6] and Oh and Lee [18] studied the change point test for the GARCH models. In addition, Bai [2], Horváth and Hušková [8] and Horváth et al. [9], etc, obtained many results of the change point detection for panel data; Shi et al. [24,25] studied the graph-based change-point test, etc.

On the other hand, the concept of Negatively Associated (NA) was first introduced by Joag-Devand Proschan [13], where they presented many examples and properties of NA sequences. One can refer to the monographs by Bulinski and Shaskin [4], Prakasa Rao [21] and Oliveira [12] for more theorems as well as applications in Gaussian system, survival analysis system, etc.

Recall that a finite family $\{Z_1, Z_2, \cdots, Z_n\}$ is said to be NA if for any disjoint subsets $A$, $B$ of $\{1, 2, \cdots, n\}$, and any real coordinatewise nondecreasing functions $f$ on $R^A$, $g$ on $R^B$,

$$\mathrm{Cov}(f(Z_k, k \in A), g(Z_k, k \in B)) \leq 0.$$

A sequence of random variables $\{Z_n, n \geq 1\}$ is said to be NA if for every $n \geq 2$, $Z_1, Z_2, \cdots, Z_n$ are NA.

In this paper, we investigate the asymptotic property of CUSUM statistic of change point under NA sequences. For convenience, let $\lfloor x \rfloor$ denote the largest integer not exceeding $x$, and $\{B^0(t); t \in [0, 1]\}$ be a standard Brownian bridge. Let $\xrightarrow{d}$ mean the convergence in distribution. Inclán and Tiao [12] proposed a CUSUM statistic to test a change-point of variance as follows:

**Theorem 1.1** *Let $\{X_n, n \geq 1\}$ be a sequence of independent, identically distributed Normal random variables with $X_1 \sim N(0, \sigma^2)$ and $\sigma^2 > 0$. Then for $k = \lfloor tn \rfloor$ and $0 \leq t \leq 1$,*

$$\max_{1 \leq k \leq n} |IT_{n,k}| \xrightarrow{d} \sup_{0 \leq t \leq 1} |B^0(t)|, \quad n \to \infty, \tag{1}$$

*where $IT_{n,k} = \sqrt{\frac{n}{2}}\left(\frac{\sum_{i=1}^k X_i^2}{\sum_{i=1}^n X_i^2} - \frac{k}{n}\right)$, $1 \leq k \leq n$.*

A large value of $\max\limits_{1 \leq k \leq n} |IT_{n,k}|$ indicates the existence of a variance change, and the change-point is at $\arg\max\limits_{1 \leq k \leq n} |IT_{n,k}|$. Meanwhile, Lee and Park [16] extended (1) to an infinite order moving average processes. For more details about the change-point detection, we can refer to the books [5,7].

In view of nonnegative of $X_i^2$ in (1), we will further investigate the asymptotic distribution of $IT_n$ in (1) based on the nonnegative sequences of NA random variables. By establishing the consistency estimators for mean and covariance functions, the limit distribution of CUSUM statistic of change point is proved to be a standard Brownian bridge. The paper is organized as follows. In Section 2, we give some assumptions and main results of this paper. In Section 3, some simulation studies and a real data analysis are implemented to show the efficiency of the CUSUM statistic. Finally, the proofs of main results are presented in Section 4.

## §2 Some assumptions and main results

Let $\{Z_n, n \geq 1\}$ be a sequence of strictly stationarity nonnegative NA random variables, and $\gamma(h)$ be the covariance function of $\{Z_n, n \geq 1\}$, which is denoted as $\gamma(h) = \mathrm{Cov}(Z_1, Z_{1+h})$, for $h = 0, 1, 2, \ldots$. $\gamma(h)$ is usually unknown and estimated by the sample covariance function $\hat{\gamma}(h) = \frac{1}{n}\sum_{i=1}^{n-h}(Z_i - \bar{\mu})(Z_{i+h} - \bar{\mu})$ for $0 \leq h < n$, where $\bar{\mu} = \frac{1}{n}\sum_{i=1}^n Z_i$, $n \geq 1$. In order to establish the main results, we need the following assumptions.

**Assumption 2.1** *Assume that*

$$\sum_{h=1}^{\infty} |\gamma(h)| < \infty \tag{2}$$

*and*

$$\sigma_0^2 := \gamma(0) + 2\sum_{h=1}^{\infty} \gamma(h) > 0. \tag{3}$$

**Assumption 2.2** *Let $\{h_n, n \geq 1\}$ be a sequence of positive integers satisfying*

$$h_n \to \infty \ \text{ as } \ n \to \infty \ \text{ and } \ h_n = O(n^\rho) \ \text{ for some } \ \rho \in (0, 1/4). \tag{4}$$

Then, the estimator for $\sigma_0^2$ is given as follows

$$\hat{\sigma}_n^2 = \hat{\gamma}(0) + 2\sum_{h=1}^{h_n}\hat{\gamma}(h). \tag{5}$$

Now, we give the main results of this paper.

**Theorem 2.1**. *Let $\{Z_n, n \geq 1\}$ be a sequence of strictly stationarity nonnegative NA random variables with $EZ_1 = \mu > 0$, $Var(Z_1) = \sigma^2 > 0$ and $EZ_1^4 < \infty$. Suppose that the Assumptions 2.1 and 2.2 are satisfied. Then, we have*

$$Var(\bar{\mu} - \mu) = O(n^{-1}) \tag{6}$$

*and*

$$\lim_{n\to\infty} E|\hat{\sigma}_n^2 - \sigma_0^2| = 0. \tag{7}$$

By Theorem 2.1, the limit distribution for the CUSUM statistic is presented as follows.

**Theorem 2.2**. *Let the conditions of Theorem 2.1 hold true. For $1 \leq k \leq n$, denote*

$$T_{nk} = \frac{\bar{\mu}\sqrt{n}}{\sqrt{\hat{\sigma}_n^2}}\Big(\frac{\sum_{i=1}^k Z_i}{\sum_{i=1}^n Z_i} - \frac{k}{n}\Big),$$

*where $\bar{\mu}$ and $\hat{\sigma}_n^2$ are defined in (6) and (7), respectively. If $t \in [0,1]$ and $k = \lfloor nt \rfloor$, then*

$$\max_{1\leq k\leq n} |T_{nk}| \xrightarrow{d} \sup_{0\leq t\leq 1} |B^0(t)| \tag{8}$$

*as $n \to \infty$.*

**Remark 2.1**. Theoretically, it is easy to obtain the consistency of mean estimator $\bar{\mu}$ (see (6)) but difficult to establish the consistency of $\hat{\sigma}_n^2$ in (7) based on the auto-covariance function estimator $\hat{\gamma}(h)$. In this paper, we use the truncation method and the covariance inequality of NA sequence (see Lemma 3.1 of Roussas [21]) to obtain the moment consistency of $\hat{\sigma}_n^2$ in Theorem 2.1. Then, the limit distribution for the CUSUM statistic $\max\limits_{1\leq k\leq n} |T_{nk}|$ is presented in Theorem 2.2. By (8), it is easy to establish (1) in Theorem 1.1 obtained by Inclán and Tiao [12]. So, Theorem 2.2 extends the result in the case of normal sequence to the dependent setting of NA sequences. In Section 3, some simulations are carried out to show that the empirical sizes and powers of our CUSUM statistic have a good performance. Further more, we apply our method and the results by Inclán and Tiao [12] to detect a change-point of variances for the returns of log daily prices of Dow Jones Industrial (DJI) index which caused by COVID-19 pandemic in 2020.

## §3 Simulation studies and a real data analysis

### 3.1 Simulations

In this subsection, we carry out some simulations to show the empirical sizes and powers for the CUSUM statistic $\max_{1\leq k\leq n} |T_{nk}|$ in (8). For convenience, if $X$ and $Y$ have the same the distribution, we denote it by $X \stackrel{d}{=} Y$. Let $k^*$ be a change-point such that

$$Y_j \stackrel{d}{=} N(0, \sigma_1^2), \quad j = 1, 2, \ldots, k^*, \quad Y_j \stackrel{d}{=} N(0, \sigma_2^2), \quad j = k^* + 1, \ldots, n, \tag{9}$$

and

$$\text{Cov}(Y_i, Y_j) = \rho, \quad \forall\ i \neq j, \tag{10}$$

where $\rho$ is some constant in $(-1, 0]$. Let $x^+ = \max(x, 0)$ and $x^- = \max(-x, 0)$. By Joag-Dev and Proschan [13], it can be seen that $\{Y_1, Y_2, \ldots, Y_n\}$ is a NA sequence. In addition, we obtain that $\{Y_1^-, Y_2^-, \ldots, Y_n^-\}$, $\{Y_1^+, Y_2^+, \ldots, Y_n^+\}$, $\{(Y_1^-)^2, (Y_2^-)^2, \ldots, (Y_n^-)^2\}$ and $\{(Y_1^+)^2, (Y_2^+)^2, \ldots, (Y_n^+)^2\}$ are nonnegative NA sequences. For simplicity, we do the simulations by 10000 replications and for the case $Z_1 = (Y_1^+)^2$, $Z_2 = (Y_2^+)^2$, $\ldots, Z_n = (Y_n^+)^2$, where $Y_1$, $Y_2$, $\ldots, Y_n$ are satisfying (9), (10) for $\rho = -n^{-2}$ ($\rho = -n^{-1.1}$) and $k^* = \lfloor \frac{n}{2} \rfloor$, $n \geq 2$. Let the null hypothesis be $H_0$: $\sigma_1^2 = \sigma_2^2$ and the alternative hypothesis be $H_1$: $\sigma_1^2 \neq \sigma_2^2$. For the significance level $\alpha = 0.05$, if $\max_{1 \leq k \leq n} |T_{nk}| > R^* = 1.358$, then we reject the null hypothesis and conclude that there is a change-point at $\hat{k}^* = \arg\max_{1 \leq k \leq n} |T_{nk}|$ (see Inclán and Tiao [12]). Consequently, for the significance level $\alpha = 0.05$, we take $h_n = \lfloor n^{1/5} \rfloor$ in (4) and obtain the empirical sizes and powers for the estimator $T_n$ in the following Table 1.

Table 1. Empirical sizes and powers for CUSUM statistic $\max_{1 \leq k \leq n} |T_{nk}|$.

| $\rho$ | $k^*$ | $n$ | size $\sigma_1^2 = \sigma_2^2 = 1$ | power $\sigma_1^2 = 1$, $\sigma_2^2 = 4$ |
|---|---|---|---|---|
| $-n^{-2}$ | $\lfloor \frac{n}{2} \rfloor$ | 300 | 0.0317 | 0.5183 |
| $-n^{-2}$ | $\lfloor \frac{n}{2} \rfloor$ | 600 | 0.0394 | 0.8875 |
| $-n^{-2}$ | $\lfloor \frac{n}{2} \rfloor$ | 900 | 0.0389 | 0.9814 |
| $-n^{-1.1}$ | $\lfloor \frac{n}{2} \rfloor$ | 300 | 0.0337 | 0.5156 |
| $-n^{-1.1}$ | $\lfloor \frac{n}{2} \rfloor$ | 600 | 0.0380 | 0.8900 |
| $-n^{-1.1}$ | $\lfloor \frac{n}{2} \rfloor$ | 900 | 0.0383 | 0.9842 |

By Table 1, we can see that, the differences of empirical sizes are smaller than 0.05 and the empirical powers go to 1 as the sample size $n$ increasing. Meanwhile, under $H_1$: $\sigma_1^2 = 1$ and $\sigma_2^2 = 4$, we obtain the histograms of estimator $\hat{k}^* = \arg\max_{1 \leq k \leq n} |T_{nk}|$ for $k^* = \lfloor \frac{n}{2} \rfloor$, $\rho = -n^{-1.1}$ and $n = 300, 600, 900$ in Fig 1.

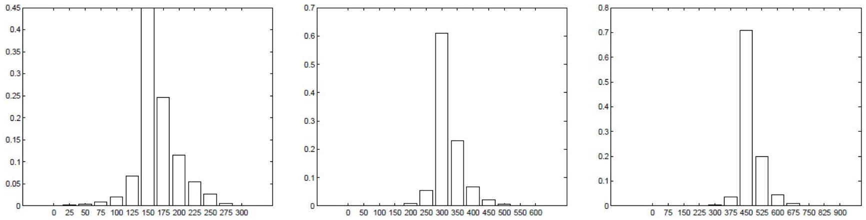

Figure 1. Histograms of $\hat{k}^* = \arg\max_{1 \leq k \leq n} |T_{nk}|$ with different sample $n = 300, 600, 900$.

By histograms in Fig 1, the percentage of $\hat{k}^*$ for $k^* = \lfloor \frac{n}{2} \rfloor$ is increasing as $n$ increasing.

## 3.2 A real data analysis

In this subsection, we apply our method and the results by Inclán and Tiao [12] to analysis the average returns of Dow Jones Industrial (DJI) index. Let $P_t$ is the price of DJI of day $t \in T$, the return is defined as $r_t = \log P_t - \log P_{t-1}$. The left of Fig 2 shows the average of log daily prices of DJI from May 2019 to March 2020 with sample sizes 232. Similarly, the right of Fig 2 shows the average of log daily prices of DJI with 31 daily returns.

By Figure 2, it looks like that there is a change-point of variance around day 200 (February 13, 2020) for the returns of log daily prices of DJI. By taking $n = 231$ and $h_n = \lfloor n^{1/5} \rfloor$, we calculate the value $\max_{1 \leq k \leq n} |T_{nk}|$ in (8), where $Z_i$ is replaced by $(r_i - \bar{r})^2$. Then $\max_{1 \leq k \leq n} |T_{nk}|$

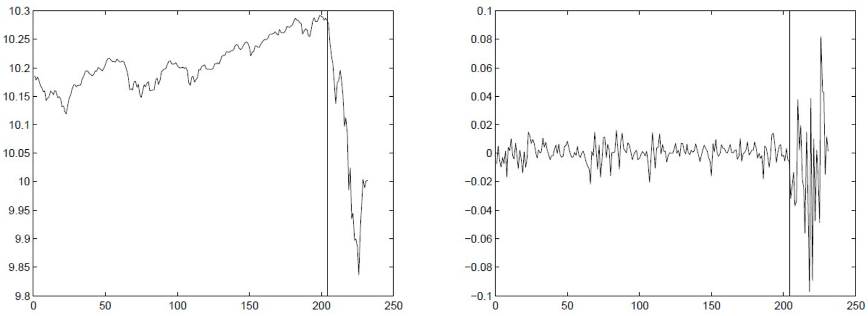

Figure 2. Log daily prices of DJI and returns of log daily prices of DJI from May 2019 to March 2020.

$= 1.6441 > 1.358$ and $\arg\max_{1\le k\le n}|T_{nk}| = 204$. Consequently, by the significance level $\alpha = 0.05$, we conclude that there is a change-point of variance for the returns $r_i$ and the change-point is at day 204. Since the method of Inclán and Tiao [12] was only used to test change-point of variance, here we also calculate the value $\max_{1\le k\le n}|IT_{nk}|$ in (1), where $X_i$ is replaced by $(r_i - \bar{r})$. Then $\max_{1\le k\le n}|IT_{nk}| = 7.7497 > 1.358$ and $\arg\max_{1\le k\le n}|IT_{nk}| = 204$. Both methods have detected the same change-point location at day 204. However, it should be pointed out that our method is not only used to detect the change-point of variance but also can be used to detect the change of nonnegative parameter. On the other hand, we find that the change-point location day 204 (February 20, 2020) is at the early stage of COVID-19 pandemic. As time goes on, the COVID-19 pandemic has caused an obviously catastrophic result to the global economy. Therefore, people all over the world should unite to defeat the COVID-19 pandemic, then humanity will finally overcome this epidemic.

## §4 Proofs of main results

For convenience, let $C, C_1, C_2, \ldots$ be some positive constants which are independent of $n$. In addition, $\Rightarrow$ denotes the weak convergence under the Skorohod topology.

**Proof of Theorem 2.1**. Obviously, by Lemma 4.2, it is easy to have that

$$\mathrm{Var}(\bar{\mu}-\mu) = \frac{1}{n^2}\mathrm{Var}(\sum_{i=1}^{n}(Z_i - EZ_i)) = O(\frac{1}{n}), \tag{11}$$

which completes the proof of (6).

Next, we will prove (7). From (3) to (5), it follows

$$E|\hat{\sigma}_n^2 - \sigma_0^2| \le E|\hat{\gamma}(0)-\gamma(0)| + 2\sum_{h=1}^{h_n} E|\hat{\gamma}(h)-\gamma(h)| + 2\sum_{h=h_n+1}^{\infty}|\gamma(h)| := L_1 + L_2 + L_3. \tag{12}$$

First, we consider $L_1$. Obviously, $\hat{\gamma}(0)-\gamma(0) = \frac{1}{n}\sum_{i=1}^{n}(Z_i^2 - EZ_i^2) - (\bar{\mu}-\mu)^2 - 2\mu(\bar{\mu}-\mu)$. For $x \ge 0$, the function $f(x) = x^2$ is increasing. Then for the nonnegative of $Z_i$, $\{Z_1^2, \ldots, Z_n^2\}$ is also a strictly stationarity NA sequence. So by Theorem 2 of Shao [22] with $EZ_1^4 < \infty$,

$$L_1 \le \Big(E\Big|\frac{1}{n}\sum_{i=1}^{n}(Z_i^2 - EZ_i^2)\Big|^2\Big)^{1/2} + E(\bar{\mu}-\mu)^2 + 2|\mu|(E(\bar{\mu}-\mu)^2)^{1/2} = O(n^{-1/2}). \tag{13}$$

Second, we consider $L_2$. Since $\hat{\gamma}(h) = \frac{1}{n}\sum_{i=1}^{n-h} Z_i Z_{i+h} - 2(\bar{\mu})^2 + \frac{n-h}{n}(\bar{\mu})^2 + \frac{\bar{\mu}}{n}\sum_{i=n-h+1}^{n} Z_i + \frac{\bar{\mu}}{n}\sum_{i=1}^{h} Z_i$ and $\gamma(h) = EZ_1Z_{1+h} - \mu^2$, then it can be seen that

$$\begin{aligned}\hat{\gamma}(h) - \gamma(h) &= \frac{1}{n}\sum_{i=1}^{n-h}(Z_iZ_{i+h} - EZ_iZ_{i+h}) - \frac{1}{n}\sum_{i=n-h+1}^{n} EZ_iZ_{i+h} - (\frac{h}{n}+1)(\bar{\mu}-\mu)^2 \\ &\quad -2\mu(\bar{\mu}-\mu) + \frac{\bar{\mu}-\mu}{n}\sum_{i=n-h+1}^{n}(Z_i - EZ_i) + \frac{\bar{\mu}-\mu}{n}\sum_{i=1}^{h}(Z_i - EZ_i) \\ &\quad +\frac{\mu}{n}\sum_{i=n-h+1}^{n}(Z_i - EZ_i) + \frac{\mu}{n}\sum_{i=1}^{h}(Z_i - EZ_i) + \frac{h\mu^2}{n} := \sum_{i=1}^{9} J_{hi}. \qquad (14)\end{aligned}$$

Therefore, it follows

$$L_2 \le C_1 \sum_{h=1}^{h_n}\sum_{i=1}^{9} E|J_{hi}|. \qquad (15)$$

For $1 \le i \le n$, denote $Z_i' = Z_i I(Z_i \le n^{1/4}) + n^{1/4} I(Z_i > n^{1/4})$, $Z_i'' = Z_i I(Z_i > n^{1/4}) - n^{1/4} I(Z_i > n^{1/4})$. In view of $Z_i = Z_i' + Z_i''$, we have that

$$\begin{aligned}\frac{1}{n}\sum_{i=1}^{n-h}(Z_iZ_{i+h} - EZ_iZ_{i+h}) &= \frac{1}{n}\sum_{i=1}^{n-h}(Z_i'Z_{i+h}' - EZ_i'Z_{i+h}') + \frac{1}{n}\sum_{i=1}^{n-h}(Z_i''Z_{i+h}' - EZ_i''Z_{i+h}') \\ &\quad +\frac{1}{n}\sum_{i=1}^{n-h}(Z_i'Z_{i+h}'' - EZ_i'Z_{i+h}'') + \frac{1}{n}\sum_{i=1}^{n-h}(Z_i''Z_{i+h}'' - EZ_i''Z_{i+h}'') \\ &:= I_1 + I_2 + I_3 + I_4. \qquad (16)\end{aligned}$$

Obviously, it follows from Hölder inequality and $EZ_1^4 < \infty$ that

$$E|I_2| \le \frac{C_1(n-h)}{nn^{1/4}}(E(|Z_1|^4 I(|Z_1| > n^{1/4})))^{1/2}(E|Z_1|^2)^{1/2} \le \frac{C_2(n-h)}{n^{5/4}}.$$

So by the fact $h_n = O(n^\rho)$ and $\rho \in (0, 1/4)$, it has

$$\sum_{h=1}^{h_n} E|I_2| \le C_1 \sum_{h=1}^{h_n}\frac{n-h}{n^{5/4}} = O(n^{\rho-1/4}) = o(1). \qquad (17)$$

Similarly,

$$\sum_{h=1}^{h_n} E|I_3| = o(1). \qquad (18)$$

Meanwhile, by Hölder inequality, it has

$$\sum_{h=1}^{h_n} E|I_4| \le C_1 \sum_{h=1}^{h_n} E(|Z_1|^4 I(|Z_1| > n^{1/4})) \le C_2 \sum_{h=1}^{h_n}\frac{n-h}{n^{3/2}} = O(n^{\rho-1/2}) = o(n^{-1/4}). \qquad (19)$$

For $1 \le i \le n-h$ and $h < n$, it has $Var(Z_i'Z_{i+h}') \le E[(Z_i')^2(Z_{i+h}')^2] \le (EZ_i^4)^{1/2}(EZ_{i+h}^4)^{1/2} \le CEZ_1^4 < \infty$. For $x \ge 0$ and $a > 0$, if $f(x) = xI(x \le a) + aI(x > a)$, then it has $\sup_x |f'(x)| \le 1$, *a.s.*. So, by Lemma 3.1 of Roussas [21], we obtain that for $1 \le i, j \le n-h$ and $i \ne j$, $|\text{Cov}(Z_i'Z_{i+h}', Z_j'Z_{j+h}')| \le C_1 n^{1/2}|\text{Cov}(Z_i', Z_j')| \le C_1 n^{1/2}|\text{Cov}(Z_i, Z_j)|$. Then, together with (2), we obtain that

$$\text{Var}(I_1) \le \frac{1}{n^2}\sum_{i=1}^{n-h}\text{Var}(Z_i'Z_{i+h}') + \frac{C_1}{n^2}\sum_{\substack{1\le i<j\le n-h \\ j-i \le \lfloor \log(h_n)\rfloor}} n^{1/2}\sqrt{\text{Var}(Z_i)\text{Var}(Z_j)}$$

$$+\frac{C_2}{n^2}\sum_{\substack{1\le i<j\le n-h\\ j-i>\lfloor\log(h_n)\rfloor}} n^{1/2}|\mathrm{Cov}(Z_i,Z_j)|\le C_3\frac{n-h}{n^{3/2}}[1+\lfloor\log(h_n)\rfloor],$$

which implies

$$\sum_{h=1}^{h_n}E|I_1|\le\sum_{h=1}^{h_n}\sqrt{\mathrm{Var}(I_1)}\le C_2\sum_{h=1}^{h_n}\sqrt{\frac{n-h}{n^{3/2}}\lfloor\log(h_n)\rfloor}=O(n^{\rho-1/4}\log^{1/2}n)=o(1),\qquad(20)$$

since $\rho\in(0,1/4)$. Consequently, from (16) to (20), it follows that

$$\sum_{h=1}^{h_n}E|J_{h1}|=o(1).\qquad(21)$$

Obviously,

$$\sum_{h=1}^{h_n}E|J_{h2}|\le\frac{1}{n}\sum_{h=1}^{h_n}\sum_{i=n-h+1}^{n}(EZ_i^2)^{1/2}(EZ_{i+h}^2)^{1/2}\le\frac{C}{n}\sum_{h=1}^{h_n}h=O(n^{2\rho-1})=o(n^{-1/2}).\qquad(22)$$

By (11), it follows

$$\sum_{h=1}^{h_n}E|J_{h3}|\le\sum_{h=1}^{h_n}(\frac{h}{n}+1)\mathrm{Var}(\bar\mu-\mu)=O(n^{\rho-1})=o(n^{-3/4})\qquad(23)$$

and

$$\sum_{h=1}^{h_n}E|J_{h4}|\le\sum_{h=1}^{h_n}2|\mu|(\mathrm{Var}(\bar\mu-\mu))^{1/2}=O(n^{\rho-1/2})=o(n^{-1/4}).\qquad(24)$$

Combining (11) with Theorem 2 of Shao [22], we get that

$$\sum_{h=1}^{h_n}E|J_{h5}|\le\frac{1}{n}\sum_{h=1}^{h_n}(\mathrm{Var}(\bar\mu-\mu))^{1/2}(E|\sum_{i=n-h+1}^{n}(Z_i-EZ_i)|^2)^{1/2}=o(n^{-9/8}).\qquad(25)$$

Similarly,

$$\sum_{h=1}^{h_n}E|J_{h6}|=O(n^{3(\rho-1)/2})=o(n^{-9/8}).\qquad(26)$$

By Lemma 4.2, it has

$$\sum_{h=1}^{h_n}E|J_{h7}|\le\frac{|\mu|}{n}\sum_{h=1}^{h_n}(E|\sum_{i=n-h+1}^{n}(Z_i-EZ_i)|^2)^{1/2}\le\frac{C_1}{n}\sum_{h=1}^{h_n}h^{1/2}=o(n^{-5/8}).\qquad(27)$$

The similarity holds true for

$$\sum_{h=1}^{h_n}E|J_{h8}|=o(n^{-5/8}).\qquad(28)$$

It is easy to have

$$\sum_{h=1}^{h_n}E|J_{h9}|\le\sum_{h=1}^{h_n}\frac{h\mu^2}{n}=O(n^{2\rho-1})=o(n^{-1/2}).\qquad(29)$$

Therefore, by (15) and (21)-(29), $L_2=o(1)$. In addition, by (2), as $h_n\to\infty$, it can be checked that $L_3=o(1)$. Consequently, together with (12) and (13), it has the result of (7). □

**Proof of Theorem 2.2**. Denote $S_n=\sum_{i=1}^n(Z_i-EZ_i)$ and $X_n(t)=\frac{S_{\lfloor nt\rfloor}}{\sqrt{n\sigma_0^2}}$ for $t\in[0,1]$, where $\sigma_0^2$ is defined by (3). Let $\{W(t);t\in[0,1]\}$ and $\{B^0(t);t\in[0,1]\}$ be standard Wiener process and standard Brownian bridge, respectively. Obviously, it can be seen that $\lim_{n\to\infty}\frac{1}{n}\mathrm{Var}(\sum_{i=1}^{n}Z_i)=\sigma_0^2>0$. By (3) and Theorem 4 of Shao [22], $X_n(t)\Rightarrow W(t)$, so $\{X_n(t)-$

$tX_n(1)\} \Rightarrow B^0(t)$ (see Page 93 of Billingsley [3]). Without loss of generality, we assume that $k = nt$, $1 \le k \le n$, since $t \in [0,1]$ and $k = \lfloor nt \rfloor$. Then

$$X_n(t) - tX_n(1) = \frac{\sqrt{n}}{\sqrt{\sigma_0^2}}\Big(\frac{1}{n}\sum_{i=1}^{n} EZ_i\Big)\Big(\frac{\sum_{i=1}^{k} Z_i}{\sum_{i=1}^{n} Z_i} - \frac{k}{n}\Big) := \frac{\sqrt{n}}{\sqrt{\sigma_0^2}}\bar{\mu}R_{nk}.$$

Therefore, by the fact $\{X_n(t) - tX_n(1)\} \Rightarrow B^0(t)$, one can obtain that $\frac{\sqrt{n}\bar{\mu}}{\sqrt{\sigma_0^2}}R_{nk} \Rightarrow B^0(t)$. By (7), it has $\hat{\sigma}_n^2 \stackrel{P}{\longrightarrow} \sigma_0^2$. So it follows $\frac{\sqrt{n}\bar{\mu}}{\sqrt{\hat{\sigma}_n^2}}R_{nk} \Rightarrow B^0(t)$. Last, by the continuous mapping theorem, we obtain $\max\limits_{1\le k\le n} \frac{\sqrt{n}\bar{\mu}}{\sqrt{\hat{\sigma}_n^2}}|R_{nk}| \stackrel{d}{\longrightarrow} \sup\limits_{0\le t\le 1}|B^0(t)|$, which implies (8) immediately. The distribution of $\sup\limits_{0\le t\le 1}|B^0(t)|$ is given in equation (9.40) of Billingsley [3]. □

## Acknowledgement

The authors are deeply grateful to editor and anonymous referees for their careful reading and insightful comments.

**Open Access** This article is licensed under a Creative Commons Attribution 4.0 International License, which permits use, sharing, adaptation, distribution and reproduction in any medium or format, as long as you give appropriate credit to the original author(s) and the source, provide a link to the Creative Commons licence, and indicate if changes were made. The images or other third party material in this article are included in the articles Creative Commons licence, unless indicated otherwise in a credit line to the material. If material is not included in the articles Creative Commons licence and your intended use is not permitted by statutory regulation or exceeds the permitted use, you will need to obtain permission directly from the copyright holder. To view a copy of this licence, visit http://creativecommons.org/licenses/by/4.0/.
